# Upper extremity compartment syndrome after minor trauma: an imperative for increased vigilance for a rare, but limb-threatening complication

**DOI:** 10.1186/1754-9493-7-5

**Published:** 2013-02-07

**Authors:** Daniel A Seigerman, Daniel Choi, Derek J Donegan, Richard S Yoon, Frank A Liporace

**Affiliations:** 1 Division of Orthopaedic Trauma, Department of Orthopaedic Surgery, UMDNJ – New Jersey Medical School, 90 Bergen Street, Suite 1200, Newark, NJ, 07101, USA; 2 Division of Orthopaedic Trauma Department of Orthopaedic Surgery, NYU Hospital for Joint Diseases, 301 E 17th Street, Suite 1402, New York, NY 10003, USA

**Keywords:** Compartment syndrome, Radial head dislocation, Upper extremity, Compartment pressures

## Abstract

**Background:**

Compartment syndrome of any extremity is a limb-threatening emergency requiring an emergent surgical management. Thus, ruling out compartment syndrome is often high on the list of priorities when treating high-energy injuries and fractures. However, even in the most seemingly benign injuries, this dangerous diagnosis must always remain on the differential and suspicion must remain high.

**Case presentation:**

23-year-old factory worker presents after a low energy entrapment injury to his left forearm. Initial work-up and evaluation noted an isolated radial head dislocation with a normal physical motor and sensory exam. However, maintaining high suspicion for compartment syndrome despite serial normal physical exams, led objective compartment pressure measurement leading to definitive diagnosis. Emergent surgical intervention via compartment fasciotomies was performed, along with closed reduction and ligament repair. At 1 year follow-up, the patient was well-healed, back to work with full range of motion and not activity limitations.

**Conclusion:**

Despite a seemingly benign injury pattern, and a relatively low energy mechanism, vigilant concern for compartment syndrome following any kind of entrapment injury should initiate serial examinations and compartment pressure measurements especially in circumstances with continued swelling and inability to perform an accurate clinical assessment due to an obtunded or medicated patient.

## Background

Isolated dislocation of the radial head without associated ulnar fracture or humeroulnar subluxation in adults is an extremely rare injury. Radial head subluxation occurs commonly in children and has been called “nursemaid’s elbow.” From 1973 to 2012, only twenty-four total cases were reported in adults [1-3]. Out of these reported cases, only six were described to be anterior dislocations and only one was described to be an anterolateral dislocation [3-8]. Compartment syndrome following an isolated dislocation of radial head is also exceedingly rare. Out of the twenty-four total cases described above, only Baraza et al. reported a possible compartment syndrome that was never confirmed by compartment pressures.

Compartment syndrome, in general, caused by acute trauma most notably in settings of crush, high-energy fractures, and/or reperfusion is an extremely dangerous complication with severe clinical consequences [9,10]. In this true emergency setting, a few hours can result in devastating consequences following a missed diagnosis with permanent sensory and motor deficits. To complicate matters further, other than serial physical examination and/or compartment pressure measurements, there is no general consensus or algorithm to aid in the accurate and timely diagnosis of compartment syndrome. However, it is important to note that compartment syndrome can also occur without high-energy mechanisms, albeit rare.

Currently, hyper vigilant concern remains the most important weapon in the arsenal in diagnosis and treatment of compartment syndrome. Here, we describe an upper extremity compartment syndrome diagnosed despite an atypical presentation and physical examination not typically found in the setting of compartment compromise. Furthermore, proposed recommendations utilized in our institution as a result of this abnormal case is also provided.

## Case report

A 23-year-old right hand dominant factory worker presented to the emergency department after his left proximal forearm became trapped between two heavy pieces of machinery. On detailed questioning, entrapment did not occur in the setting of high energy, rather it occurred during an attempt to retrieve an object that had fallen between the two metal slabs. Following approximately 35 minutes, workers were able to free the patient and the patient presented to the emergency department complaining of inability to flex and extend his left elbow with associated pain. At presentation, patient denied any loss of motor function, or sensation of numbness and/or tingling in the affected extremity.

On initial physical examination, the proximal forearm was swollen, minimally tense and tender to palpation. The patient exhibited limited passive range of motion about the left elbow ranging from 30 to 90 degrees of flexion and extension, and a 30-degree arc of pronation/supination. Most importantly, there was no pain with passive stretch at the wrist and fingers, and with associated full, painless active range of motion. The patient was sensory intact to two-point discrimination at 5 mm at all distal distributions with a palpable distal radial pulse.

**Figure 1:**
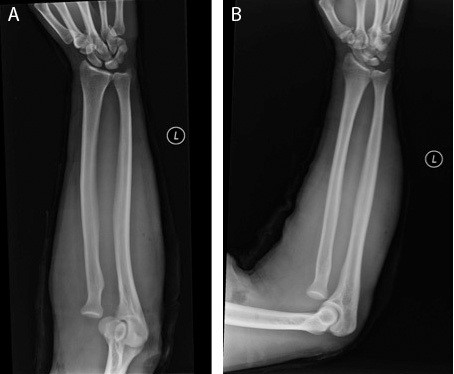
A-B. A) AP and B) lateral view of injury films noting left radial head dislocation.

Initial radiographic examination demonstrated an anterolateral radial head dislocation (Figure 1A-B). Two unsuccessful closed reduction attempts under conscious sedation were performed. The decision to measure the forearm compartments was made based on several factors. First, although a low energy mechanism, the entrapping nature of the injury was the major component in proceeding with needle monitoring. Additional factors included persistent swelling, especially following unsuccessful manipulation attempts coupled with a slightly obtunded state secondary to conscious sedation. These factors, despite a normal physical exam were the major components in moving forward with needle monitoring. A standard Stryker compartment pressure-monitoring needle (Stryker Inc., Mahwah, NJ, USA) was utilized. The patient’s diastolic blood pressure at this time was measured to be 61 mmHg. Compartment pressure readings of the mobile wad, extensor, and flexor compartments were 57 mmHg, 35 mmHg, and 38 mmHg respectively. All compartments demonstrated a delta-p greater than 30 mmHg confirming the diagnosis of compartment syndrome.

**Figure 2:**
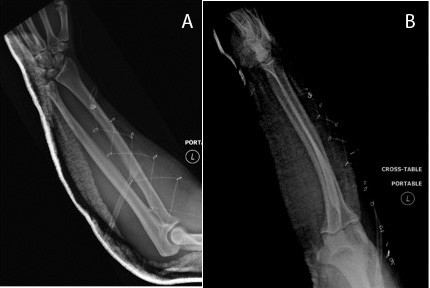
A-B. A) AP and B) lateral view s/p compartment release and open reduction.

The patient was then taken emergently to the operating room for open reduction and fasciotomy. A lateral incision over the mid-aspect of the radius starting distally at the distal third junction of the forearm carried proximally to the lateral epicondyle and continued three fingerbreadths proximal to the lateral epicondyle in line with the lateral aspect of the humerus was made. Individual fascial incisions of all muscles in the extensor compartment were performed using Metzenbaum scissors. The musculature of the mobile wad was then released as well. At this point, the interval between the extensor carpi ulnaris and anconeus was developed, where the dislocated radial head was encountered. The annular ligament was found to be partially avulsed and the radial head was button holed through the annular ligament. The incarcerated annular ligament was freed and the radial head was easily reduced (Figure 2A-B). Subsequent repair of the annular ligament as well as the interval between the anconeus and extensor carpi ulnaris was performed. At this point, the compartments were again measured with a Stryker compartment monitoring needle. The diastolic blood pressure was measured to be 54 mmHg. Flexor, extensor, and mobile wad compartments were found to be 6, 5, and 9 mmHg respectively. Delta-p values were 48, 49, and 45 mmHg respectively. The skin wound was left open over the mobile wad, and the wound was covered with a Vacuum Assisted Closure (VAC) (Kinetic Concepts Inc., San Antonio, TX, USA) dressing. All remaining wounds were closed primarily.

The patient was taken back to the operating room three days later for irrigation and debridement, as well as VAC dressing change where all muscle tissue was found to be viable. Three days later, the patient was taken for split thickness skin grafting over the mobile wad, using the ipsilateral thigh as the donor site. The patient was immobilized for two weeks post-operatively to protect the soft tissues as well as the annular ligament repair, and was progressed to full range of motion as tolerated two weeks from his final procedure. He was enrolled in a physical therapy program for range of motion, strengthening, and stretching of the left elbow and left upper extremity.

**Figure 3:**
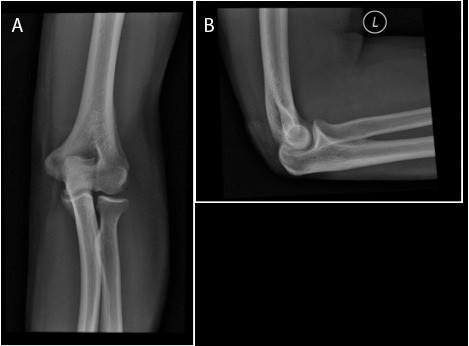
A-B. A) AP and B) lateral view at one-year follow-up.

**Figure 4:**
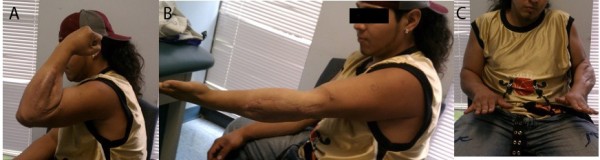
A-C). Clinical photos at one-year follow-up noting full active range of motion without deficit.

Final follow-up at 1 year noted radiographically confirmed, concentric reduction of the left elbow (Figure 3A-B). He has 5/5 strength with elbow flexion and extension from 0–160 degrees, and can both actively pronate and supinate to 80 degrees (Figure 4A-C). He has returned to work at full duty as a laborer and has no functional limitations.

We report a unique case of a patient developing a compartment syndrome after a low energy entrapment with subsequent anterolateral dislocation of the radial head. This case highlights the importance of identifying the mechanism of injury when characterizing and treating bony injuries., while maintaining vigilance for compartment syndrome despite a low energy mechanism. Vigilance to a detailed history and accurate account of the injury were imperative in this case, and although a low energy mechanism, entrapment of his extremity drove us to the pressing diagnosis of compartment syndrome, where radiographs and physical examination alone may not have sufficed.

Various mechanisms of injury have been described for anterior dislocation of the radial head: forceful biceps contraction, hyper-pronation associated with slight elbow flexion and varus strain from a fall, direct blow to the posterior aspect of a semiflexed elbow, and hyperextension in supination [4-7,11]. In our case, the mechanism was entrapped forearm, which has never been described.

Most acute cases of isolated radial head dislocation can be closed reduced, but functional outcomes are not well documented in these cases and there are no established treatment guidelines. Unsuccessful closed reduction of anterior dislocations is due to a torn and/or incarcerated annular ligament/LCL complex and these structures must first be released and repaired [11,12]. This was the case in our patient as two unsuccessful closed reduction attempts were performed. Intraoperatively, the dislocated radial head was button holed through the annular ligament and the incarcerated annular ligament was freed and repaired. A variety of other procedures have also been proposed such as radial head excision, Kirschner-wire fixation of radiohumeral joint, and tension band wiring if the annular ligament/LCL complex is damaged irreparably or if there is instability despite repair of the annular ligament/LCL complex [4,7,8,12]. In our case, the patient did well after a repair of the annular ligament and did not report any elbow instability in post-operative follow-up.

In a case with anterior dislocation of the radial head by Baraza et al., compartment syndrome was not confirmed by the measurement of compartment pressures. A pre-emptive fasciotomy was prompted by severe swelling and tightness of the forearm and the underlying muscle was dusky but bleeding and contractile [4]. In our case, compartment syndrome was confirmed by measuring compartment pressures with a Stryker needle despite minimal clinical symptoms of compartment syndrome. Clinically, the decision to obtain needle pressures was the definitive diagnostic measure, especially in a patient in an obtunded state secondary to conscious sedation. High clinical suspicion and vigilance led to the use of the needle pressure measurements, leading to an eventual good clinical outcome.

Our clinical scenario was confounded by the relative lack of the cardinal features often associated with compartment monitoring on physical exam, namely pain, pallor, pulseslessness, parasthesia, pressure, poikilothermia, and paralysis [13,14]. All of these symptoms, lack considerably sensitivity in the diagnosis of compartment syndrome, but offer high specificity, specially when multiple symptoms are present [15]. Herein lies the major controversy in the management of compartment syndrome as a fine line exists between prophylactic, morbid release of compartments, or waiting for definitive symptom presentation, which can regrettably become a decision made too late in the process and yield suboptimal clinical results. Thus, in situations like these, where physical examination plays a detrimental role in diagnosis, high suspicion based on mechanism of injury and subsequent use of a compartment measuring needle may prove of paramount importance.

## Conclusion

In conclusion, high clinical suspicion, obtained from a detailed history regarding mechanism of injury should be the major driving force in the diagnostic decision-making processes involving compartment syndrome. While certain scenarios, such as high energy injuries with associated fractures may offer a more obvious picture to rule out compartment syndrome, all entrapment injuries, should evoke a vigilant algorithm to confirm or rule out compartment syndrome. When faced with an obtunded or sedated patient, suspicion should remain high based on mechanism alone and definitively monitored with needle measurements when needed. The clinical scenario described has led to the more consistent and liberal use of needle compartment pressure measurements, especially in high energy and crush injury scenarios, but also most importantly in settings of extremity entrapment, even with low energy.

## Consent

Written informed consent was obtained from the patient for publication of this Case report and any accompanying images. A copy of the written consent is available for review by the Editor-in-Chief of this journal.

## Competing interests

The authors have no competing interests to declare in the preparation or finalization of this manuscript.

## Authors’ contributions

DAS (Concept & design, initial draft, final approval); DC (data collection, image collection, initial draft, final approval); DJD (literature search, initial draft, concept/design, final approval); RSY (initial draft, image preparation, concept/design, final approval, revisions) and FAL (oversight, concept/design, image approval, initial draft, final approval, revisions). All authors read and approved the final manuscript.
